# Beta-Lactamase Repressor BlaI Modulates *Staphylococcus aureus* Cathelicidin Antimicrobial Peptide Resistance and Virulence

**DOI:** 10.1371/journal.pone.0136605

**Published:** 2015-08-25

**Authors:** Morgan A. Pence, Nina M. Haste, Hiruy S. Meharena, Joshua Olson, Richard L. Gallo, Victor Nizet, Sascha A. Kristian

**Affiliations:** 1 Biomedical Sciences Graduate Program, University of California San Diego, La Jolla, CA, United States of America; 2 Department of Pediatrics, School of Medicine, University of California San Diego, La Jolla, CA, United States of America; 3 Skaggs School of Pharmacy and Pharmaceutical Sciences, University of California San Diego, La Jolla, CA, United States of America; 4 Department of Dermatology, School of Medicine, University of California San Diego, La Jolla, CA, United States of America; 5 VA San Diego Healthcare System, San Diego, CA, United States of America; Institut National de la Recherche Agronomique, FRANCE

## Abstract

BlaI is a repressor of BlaZ, the beta-lactamase responsible for penicillin resistance in *Staphylococcus aureus*. Through screening a transposon library in *S*. *aureus* Newman for susceptibility to cathelicidin antimicrobial peptide, we discovered BlaI as a novel cathelicidin resistance factor. Additionally, through integrational mutagenesis in *S*. *aureus* Newman and MRSA Sanger 252 strains, we confirmed the role of BlaI in resistance to human and murine cathelidicin and showed that it contributes to virulence in human whole blood and murine infection models. We further demonstrated that BlaI could be a target for innate immune-based antimicrobial therapies; by removing BlaI through subinhibitory concentrations of 6-aminopenicillanic acid, we were able to sensitize *S*. *aureus* to LL-37 killing.

## Introduction

*Staphylococcus aureus* is a leading cause of community- and hospital-associated infections, ranging from superficial syndromes to several potentially life-threatening invasive conditions including sepsis and endocarditis [1]. Penicillin was once the drug of choice for treatment of *S*. *aureus* infections. However, penicillin-resistant strains due to beta-lactamase production were reported as early as 1942, and today over 95% of human *S*. *aureus* isolates are resistant to penicillin [2]. The beta-lactamase-resistant penicillin derivate methicillin was introduced in 1961, but the first methicillin-resistant *S*. *aureus* (MRSA) strains appeared shortly thereafter. Recent reports of *S*. *aureus* isolates with intermediate or complete vancomycin resistance may foreshadow an era in which effective treatment of *S*. *aureus* infections may become extraordinarily complicated. Therefore, new treatment measures and the identification and characterization of additional targets for anti-staphylococcal therapy are urgently needed.

**Fig 1:**
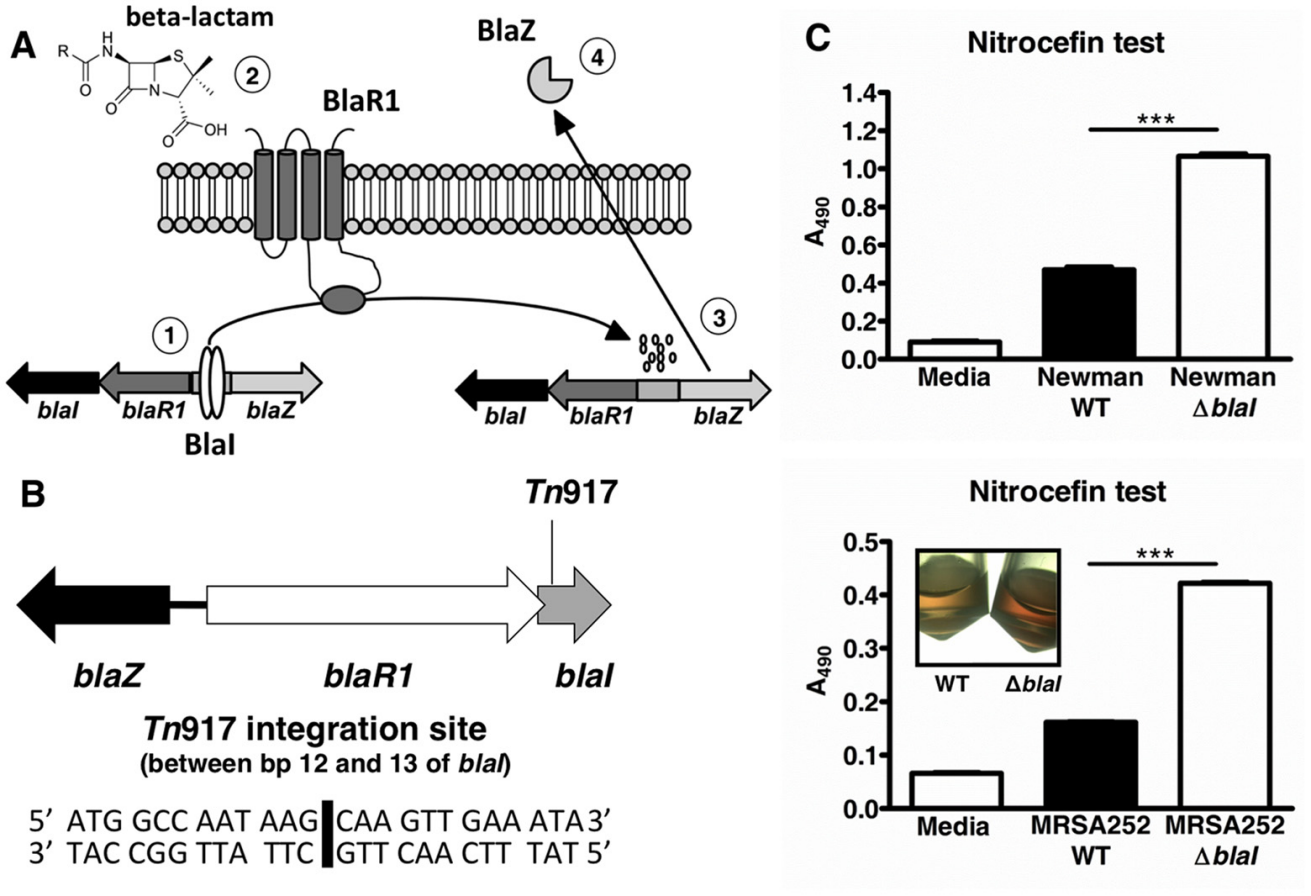
Mapping of the *Tn*917 mutant and subsequent inactivation of *blaI* leading to elevated beta-lactamase production. (A) (1) In the absence of beta-lactams, the *blaZ-blaR1-blaI* genes are repressed by BlaI. (2) When beta-lactam molecules are sensed by BlaR1, the cytoplasmatic domain of this transmembrane protein is autoproteolytically cleaved. (3) Following this event, the repressor protein BlaI is proteolytically cleaved and dissociates from its binding site, enabling transcription of the beta-lactamase-encoding gene *blaZ*. (4) Finally, the active beta-lactamase BlaZ is secreted, leading to hydrolysis of the beta-lactam molecules. (B) Organization of the *blaZ-blaR1-blaI* locus in *S*. *aureus* Newman. *blaR1* and *blaI* are located in a two-gene operon. *blaZ* is divergently transcribed. In the cathelicidin susceptible *S*. *aureus* Newman mutant G2E3, *Tn*917 integration occurred 12 bp downstream of the *blaI* start codon. (C) *S*. *aureus* Newman WT and *blaI* mutant or MRSA252 WT and *blaI* mutant were incubated with 50 μg/ml nitrocefin for 30 min at 37°C, and the absorbance at 490 nm (A_490_) was read. A higher A_490_ value reflects higher beta-lactamase activity. Mean A_490_ values ± SD of duplicates of one representative experiment of at least three performed for each strain are shown. ***, *p*<0.001. A representative picture for the MRSA252 WT and *blaI* mutant bacteria after incubation with nitrocefin are shown.

Beta-lactamase-mediated penicillin resistance in *S*. *aureus* has been thoroughly investigated [2]. The inducible *S*. *aureus* PC1 beta-lactamase is encoded by *blaZ*, and the transcription of *blaZ* is controlled by the BlaZ-BlaR1-BlaI system [3,4]. The genes for BlaZ, its repressor BlaI and the signal transducer-sensor protein BlaR1 are clustered together, either on a plasmid or within the bacterial chromosome [5]. In the absence of beta-lactam exposure, the DNA repressor BlaI represses *blaZ* by binding to the conserved DNA motif TACA/TGTA, located in the promoter region of *blaZ* [6,7]. The detection of beta-lactam molecules by BlaR1 initiates a signaling cascade, ultimately resulting in de-repression of *blaZ* (illustrated schematically in Fig 1A).

Antimicrobial peptides (AMPs) are fundamental components of mammalian innate immunity to control microbial infections and coordinate host responses to infection. The AMPs identified in humans include members of the cathelicidin, defensin, thromobicidin and histatin families. The only cathelicidin found in humans is LL-37, while a functional homolog, CRAMP, is present in mice. Bacterial pathogens have evolved countermeasures to decrease their susceptibility to host AMPs including (i) decreased affinity to AMPs through cell envelope modifications, (ii) active efflux pumps, (iii) external trapping of AMPs, (iv) production of AMP degrading proteases, and (v) interference with host AMP production [8,9]. In the last several years, it has been established that the degree of resistance to host AMPs correlates with the potential of pathogenic bacteria to cause disease. Consequently, others and we have suggested that AMP resistance factors could be targets for novel anti-bacterial drugs [10,11].

The work described in this paper provides evidence that *S*. *aureus* BlaI regulation extends beyond the scope of beta-lactamase expression, and contributes in addition to the pathogen’s resistance to cathelicidin AMPs. As a consequence, BlaI contributes to *S*. *aureus* innate immune evasion and virulence. Pharmacological ablation of BlaI by low concentrations of beta-lactams renders penicillin-resistant *S*. *aureus* more susceptible to innate immune defenses. This finding highlights a potential indirect utility of beta-lactam antibiotic treatment regimens in treatment of MRSA infections.

## Results

### Inactivation of *blaI* Leads to Increased Cathelicidin Susceptibility in *S*. *aureus*

To identify staphylococcal genes involved in cathelicidin resistance, a random mutant library of *S*. *aureus* Newman was generated by *Tn*917 transposition. Individual mutants were screened for increased susceptibility to the murine cathelicidin CRAMP. From 4,800 *Tn*917 mutants screened, 19 showed >4-fold increased sensitivity to CRAMP as compared to the wild-type (WT) strain. Putative CRAMP susceptible mutants identified in the primary screen were subjected to additional testing to confirm their phenotype by characterizing (i) their susceptibility to CRAMP in exponential and stationary phase and (ii) by demonstrating that the mutants exhibited no replication defects in growth curves compared to the WT strain in Todd Hewitt broth (THB; data not shown).

Five of the 19 CRAMP-hypersusceptible mutants mapped to varying locations within the *blaI* gene. In one of these mutants (G2E3) the transposon insertion was mapped between base pairs (bps) 12 and 13 of the beta-lactamase repressor gene *blaI*, which is part of the *blaZ-blaR1-blaI* locus. The insertion of the transposon in the *blaI* gene was further confirmed by PCRs with *blaI* and *Tn*917 specific primer pairs (data not shown). The *blaZ-blaR1-blaI* locus is not found in the published chromosome sequence of *S*. *aureus* Newman [12] and thus must be present in an extrachromosomal locus such as a plasmid. The organization of the *blaZ-blaR1-blaI* locus for several *S*. *aureus* strains is well characterized [2] and was found to be the same in *S*. *aureus* Newman as determined by PCRs. Fig 1B illustrates the organization of the *blaZ-blaR1-blaI* locus in *S*. *aureus* Newman and the location of the *Tn*917 insertion in the cathelicidin susceptible mutant G2E3. Of note, in sequenced *S*. *aureus blaZ-blaR1-blaI* loci such as that of MRSA Sanger 252 (MRSA252) [5], *blaR1* overlaps with the *blaI* gene by 11 bps. Thus, we assumed that the transposon insertion in mutant G2E3 occurred 1 bp downstream of *blaR1*. Of note, none of the CRAMP-hypersusceptible mutants mapped to the *blaZ* gene.

BlaI was confirmed as a cathelicidin resistance factor by targeted integrational mutagenesis of *blaI* and *in trans* complementation of the resulting *blaI* mutant strain in *S*. *aureus* Newman. In addition, *blaI* was inactivated in MRSA252, in which the *bla* operon is located on the bacterial chromosome [5]. The beta-lactamase activities of both genetically engineered *blaI* mutant strains were compared to those of the respective wildtype (WT) strains using nitrocefin as the test reagent. The WT strains showed significant beta-lactamase activity in the presence of the beta-lactam nitrocefin as compared to the medium only controls. Disruption of the beta-lactamase repressor *blaI* led to ~twofold elevated beta-lactamase (BlaZ) activity in both *S*. *aureus* Newman and MRSA252 *blaI* mutant strains (Fig 1C). The complemented Newman *blaI* strain also demonstrated increased beta-lactamase activity, inferring that BlaI activity may not be completely restored in that strain upon plasmid complementation (data not shown). As expected, a MRSA252 *blaZ* mutant completely lacked activity in the nitrocefin test confirming functional inactivation of the beta-lactamase BlaZ (data not shown).

Additionally, we tested the minimum inhibitory concentration (MIC) [13] of penicillin G against the strains as a second readout of beta-lactamase activity. Compared to the WT strains, we observed a slight, but reproducible increase in the MICs of *blaI* mutants in both the Newman (64 μg/ml for WT vs. ≥128 μg/ml for *blaI* mutant) and MRSA252 (64 μg/ml for WT vs. 128 μg/ml for *blaI* mutant) backgrounds. The complemented Newman *blaI* strain had an MIC similar to the mutant (≥128 μg/ml), additional evidence that BlaI activity may not be completely restored. These results indicated that the expression of the beta-lactamase BlaZ was de-repressed through inactivation of the *blaZ* repressor BlaI. Furthermore, to determine if BlaI played a role in resistance to the anti-staphylococcal antibiotic daptomycin, we tested the susceptibility of the Newman strains using a standard Etest. The *blaI* strain had a slightly decreased MIC (0.125 μg/ml) compared to the WT (0.250 μg/ml) and complemented mutant (0.250 μg/ml) strains.

**Fig 2:**
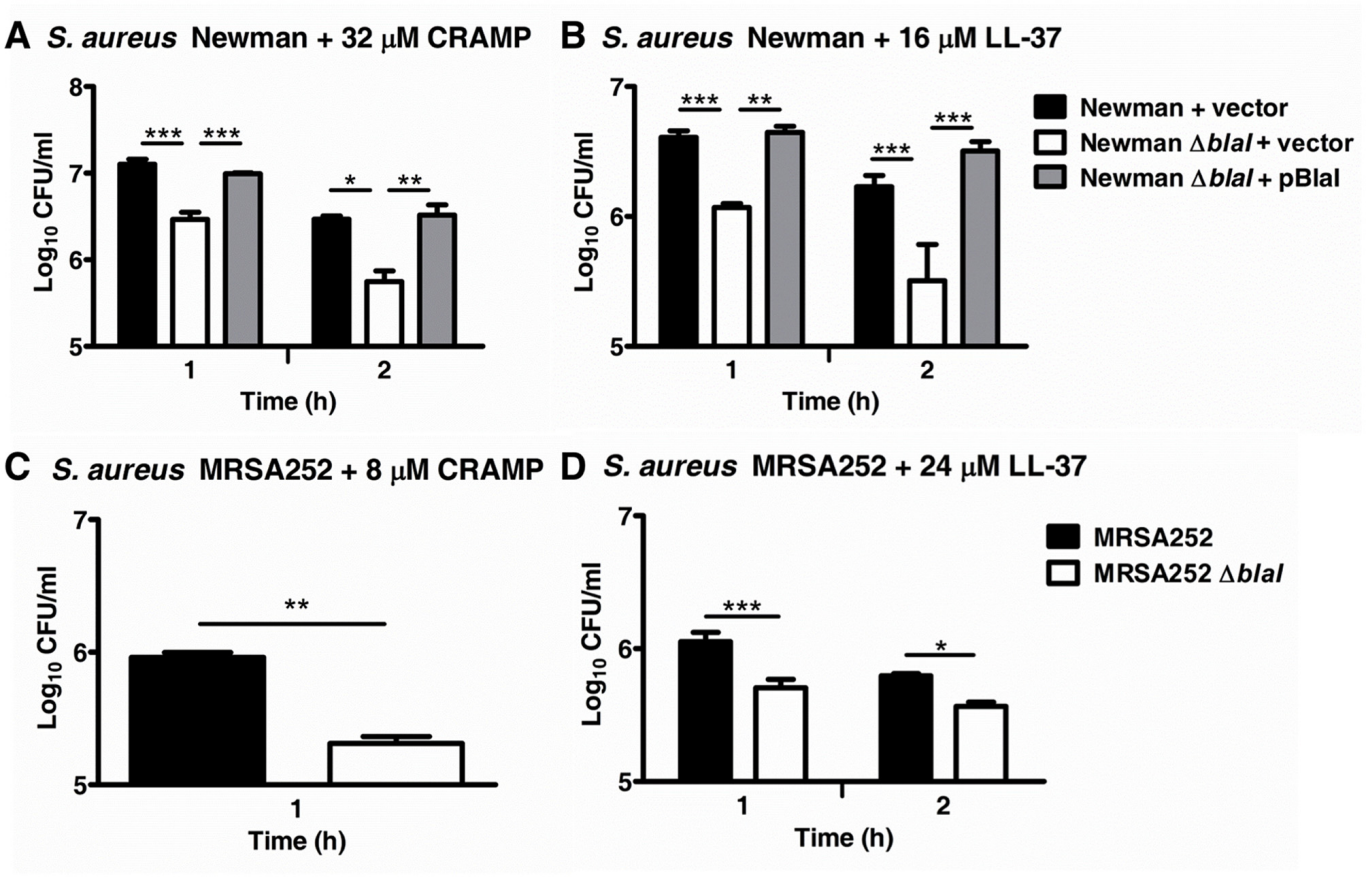
Effect of *blaI* on the cathelicidin susceptibility of *S*. *aureus*. (A, B) *S*. *aureus* Newman and (C, D) MRSA252 WT, *blaI* mutant or complemented mutant strains were incubated with CRAMP or LL-37 and the numbers of surviving CFUs were determined at the indicated time points. Samples were run in triplicate, and average CFU/mL values ± SD for one representative experiments of at least two performed for each data set is shown on a log scale. *, *p*<0.05; **, *p*<0.01, ***, *p*<0.001.

We next performed killing kinetics with cathelicidin AMPs. Compared to their respective *S*. *aureus* Newman and MRSA252 WT strains, the *blaI* mutants were found to be more susceptible to the murine cathelicidin CRAMP and human LL-37 with their CFU concentrations being approximately 0.5 logs lower compared to the respective WT strains after 1–2 hours of co-incubation with the cathelicidins (Fig 2A–2D). The difference in AMP susceptibility between the Newman and MRSA252 WT and their respective *blaI* mutants was observed for both stationary and exponential phase cells (data not shown) indicating that the phenotype was growth phase-independent. Despite the fact that expression of BlaI *in trans* on plasmid pBlaI did not fully abolish the increased beta-lactamase activity (see above), the *S*. *aureus* Newman *blaI* mutant expressing pBlaI showed WT levels for both CRAMP and LL-37 resistance indicating functional complementation of *blaI* in terms of cathelicidin susceptibility (Fig 2A and 2B). Additionally, no differences in cathelicidin killing as compared to the MRSA252 WT strain were observed for a *blaZ* mutant in this strain background (data not shown) further ensuring that the cathelicidin susceptibility phenotype of the *blaI* mutant was specific to the *blaI* gene.

### BlaI Promotes Survival of *S*. *aureus* in Human Whole Blood and Virulence in Murine Infection Models

**Fig 3:**
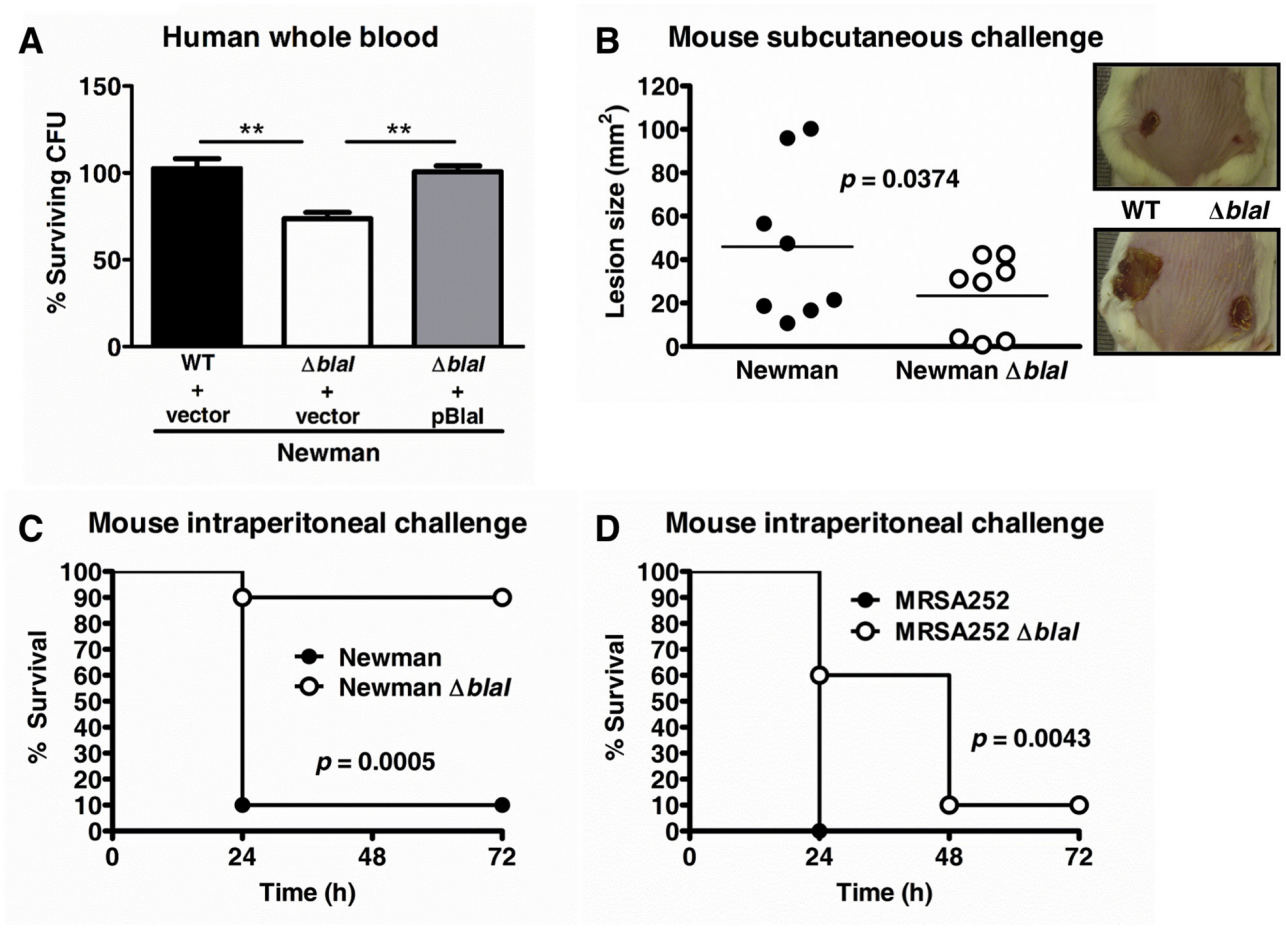
BlaI contributes to survival in human whole blood and virulence *in vivo*. (A) *S*. *aureus* Newman WT with empty complementation vector pDC123, the *blaI* mutant with pDC123, and the complemented *blaI* mutant strain were incubated for 1 h in human whole blood and CFU numbers enumerated. Samples were run in triplicate and data were plotted as the average percentage ± SD for each strain as compared to the initial inocula. A representative experiment of three performed is shown. **, *p*<0.01. (B) CD-1 mice (*n* = 8) were injected subcutaneously on one flank with *S*. *aureus* Newman WT and on the opposite flank with *blaI* mutant bacteria, and lesion sizes were monitored for 7 days. The lesions for each individual mouse at Day 7 are plotted and the average value indicated. Overall, the *blaI* mutant lesions were significantly smaller compared to the WT (*p*<0.04; paired t-test). (C-D) Survival of CD-1 mice (*n* = 10) after intraperitoneal infection with (C) 1 x 10^6^ CFU of *S*. *aureus* Newman WT or Newman *blaI* mutant or (D) 6 x 10^8^ CFU of MRSA252 or MRSA252 *blaI* mutant. Survival was monitored for 3 days. The survival for Newman or MRSA252 *blaI* mutant strain infected mice was significantly higher than for the WT infected strains as assessed by log-rank (Mantel Cox) test; the *p* values are shown in the respective graphs.

To establish whether the *blaI*-mediated cathelicidin resistance could lead to increased survival of *S*. *aureus* in the host, we performed killing assays in human whole blood and compared the virulence of WT and *blaI* mutant bacteria in murine abscess and septicemia models. As shown in Fig 3A, the *S*. *aureus* Newman *blaI* mutant was killed significantly better by human whole blood than the WT and complemented strains. In a murine skin abscess model, lesions formed by WT *S*. *aureus* Newman were significantly larger than those created by the *blaI* mutant (Fig 3B), and in murine sepsis models, virulence of the *blaI* mutant was found strongly attenuated in *S*. *aureus* Newman (Fig 3C) and moderately impaired in MRSA252 (Fig 3D). Taken together, the human whole blood *ex vivo* and the mouse *in vivo* data suggested that BlaI contributes to the pathogenic potential of *S*. *aureus*.

### The Surface Charge of *S*. *aureus* Is Not Altered by *blaI* Inactivation

**Fig 4:**
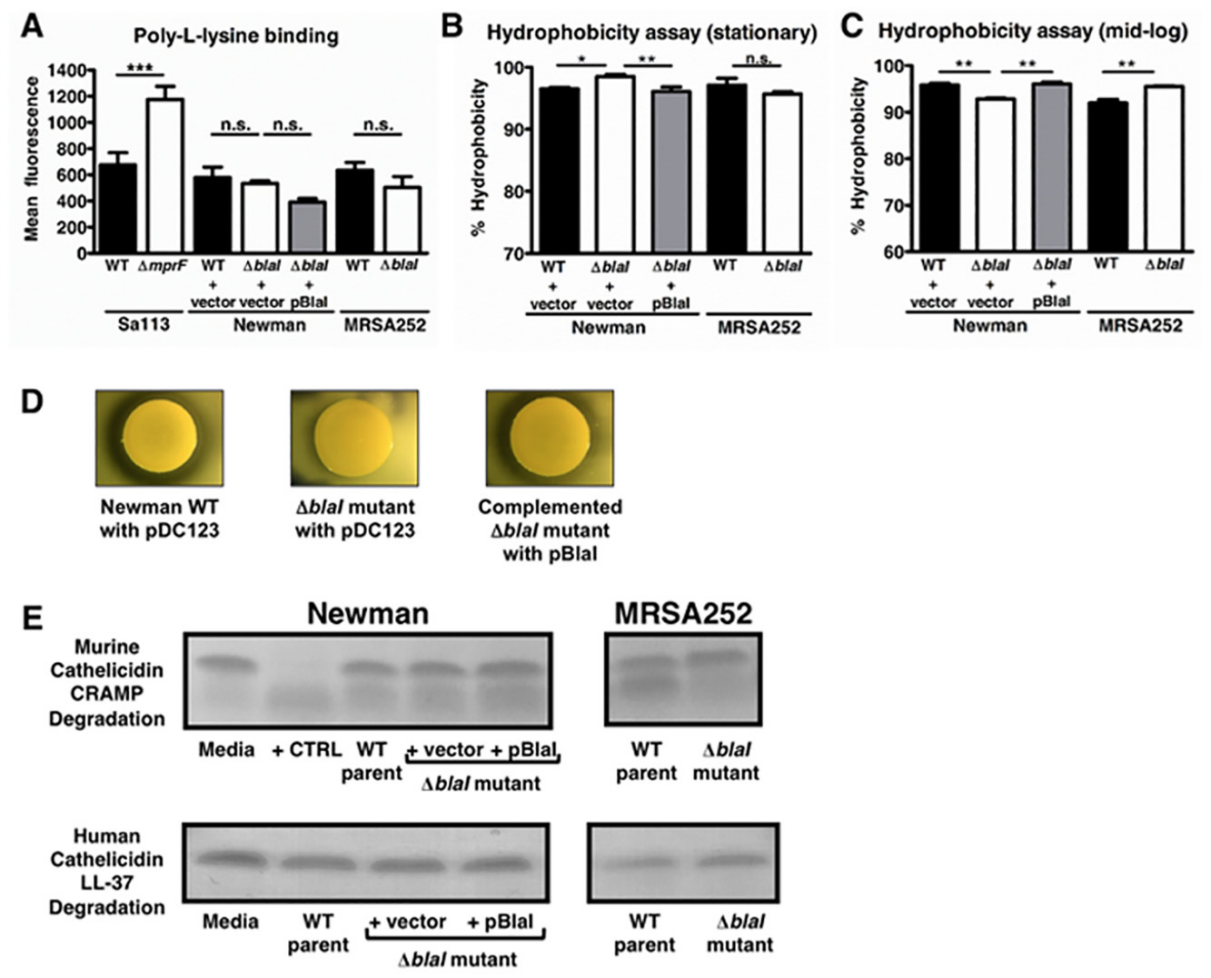
Potential mechanisms of cathelicidin resistance. (A) Surface charge of *S*. *aureus* Newman and MRSA252 strains was compared by poly-L-lysine binding. The *S*. *aureus* strain Sa113 and an isogenic *mprF* mutant with increased negative surface charge were used as controls. (B, C) Hydrophobicity was measured using a modified version of the MATH (microbial adhesion to hydrocarbons) assay. *, *p*<0.05; **, *p*<0.01; ***, *p*<0.001; n.s., not significant. (D) Proteolytic activity of Newman WT + pDC123, the *blaI* mutant + pDC123 and the *blaI* mutant complemented with pBlaI on skim milk agar plates. Clearance zones around colonies indicate secreted protease activity. (E) Degradation of CRAMP or LL-37 by overnight cell-free supernatants analyzed by SDS-PAGE.

After establishing that BlaI contributes to cathelicidin resistance, whole blood survival and the virulence potential of *S*. *aureus*, we aimed to determine the mechanism through which BlaI exerts these actions. A common AMP resistance mechanism among Gram-positive and Gram-negative bacteria is to reduce their net negative surface charge through cell envelope modifications in order to repel positively charged AMPs [8]. However, the Newman and MRSA252 WT and their respective *blaI* mutant strains showed no significant difference in surface charge as assessed by poly-L-lysine-FITC binding (Fig 4A). A strain pair of *S*. *aureus* Sa113 and an isogenic *mprF* negative mutant served as controls, where the mutant strain showed increased Poly-L-lysine binding as expected from the literature [14].

### Hydrophobicity Is Modestly Affected by Inactivation of *blaI*

Another mechanism by which bacteria resist AMPs is through increased hydrophobicity of their cell membrane [15]. To test if there was a difference in hydrophobicity between WT and *blaI* mutant strains, we utilized a version of the microbial adhesion to hydrocarbon assay. We observed small, though significant, differences in hydrophobicity between the Newman WT and *blaI* mutant strains in stationary phase and between both the Newman and MRSA252 WT and their respective *blaI* mutant strains in mid-log phase (Fig 4B and 4C). However, the differences were differing in direction based on growth phase. As mentioned above, AMP susceptibility differences between WT and *blaI* mutant strains were found to be growth phase-independent. Thus, it was unlikely that hydrophobicity was playing a major role if any in BlaI-mediated AMP resistance.

### Proteolytic Activity Is Decreased in *S*. *aureus* Newman and MRSA252 *blaI* Mutants

Proteolytic cleavage is a third mechanism bacteria utilize to resist AMPs. To test if there were differing levels of protease activity in the strains, we used skim milk agar plates. As demonstrated in the representative result in Fig 4D, colonies of the *S*. *aureus* Newman WT strain carrying the empty complementation vector pDC123 showed robust zones of clearance around them demonstrating protease activity. In contrast, the *blaI* mutant with pDC123 did not have any apparent protease activity on skim milk agar plates (Fig 4D). Since the protease activity could be restored to WT levels by expression of *blaI* in trans in the complemented mutant strains, the higher proteolytic activity of the WT strain could be attributed to the presence of functional *blaI*/BlaI (Fig 4D). MRSA252 WT and *blaI* mutant strains did not show any evident protease activity on skim milk agar plates (data not shown).

To test if there were differing levels of cathelicidin cleavage between the WT and their respective *blaI* mutant strains, we incubated overnight cell-free supernatants with CRAMP or LL-37 for 24 h and subsequently ran the samples on an SDS-PAGE gel. We did not discern any differences between the Newman WT and *blaI* mutant strains but did observe a decrease in CRAMP cleavage by the MRSA252 *blaI* mutant compared to its parental strain (Fig 4E).

### Subinhibitory Concentrations of 6-aminopenicillanic (6-APA) Renders Penicillin-Resistant *S*. *aureus* More Susceptible to Cathelicidin-Mediated Killing

**Fig 5:**
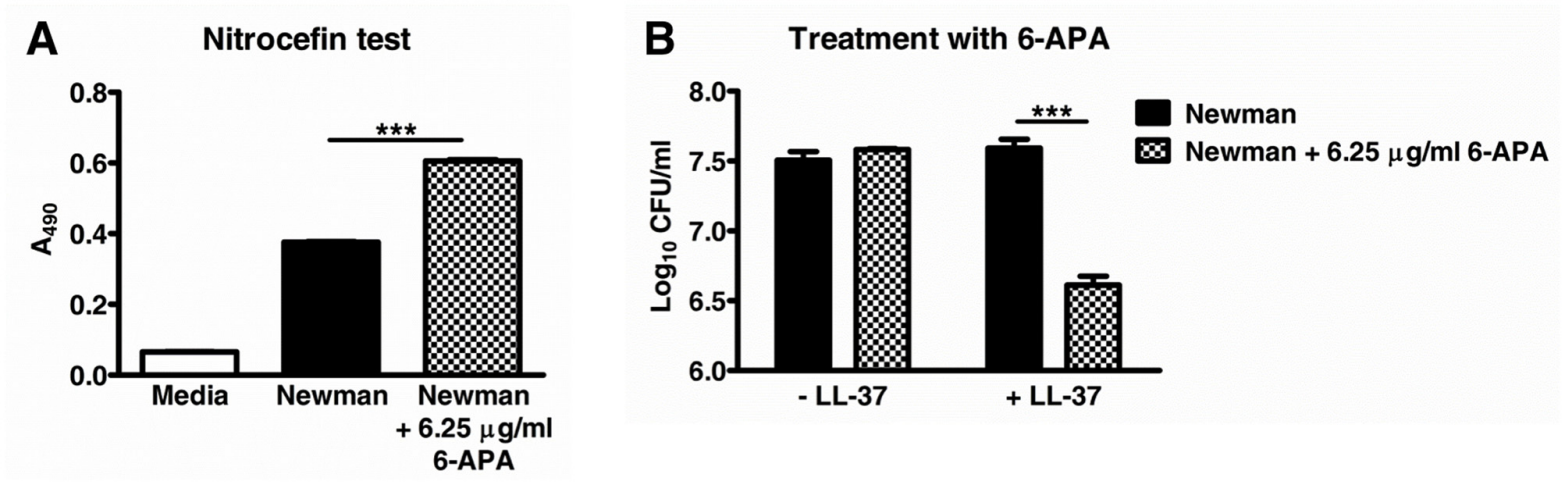
Pre-incubation with subinhibitory concentrations of the beta-lactam antibiotic 6-APA increases the beta-lactamase activity and LL-37 susceptibility of *S*. *aureus* Newman. Bacteria were incubated for 60 min at 37°C in the absence or presence of 6-APA. For an aliquot of each sample the beta-lactamase activity was subsequently determined with nitrocefin as test reagent (A). The residual bacteria were incubated with or without LL-37 for another 60 min and the surviving CFUs were quantified (B). Samples were run in triplicate and one representative experiment of three performed is shown. ***, *p*<0.001.

We hypothesized that the exposure of *S*. *aureus* Newman WT to beta-lactams would force the bacteria to remove their beta-lactamase repressor BlaI and in consequence would lead to increased susceptibility to cathelicidin AMPs. To address this possibility, *S*. *aureus* Newman was incubated with a subinhibitory concentration of 6-APA, which is the active core structure of all penicillins. Subsequently, the killing by LL-37 was tested as compared to untreated cells. As can be seen in Fig 5, subinhibitory concentrations of 6-APA led to increased beta-lactamase activity in *S*. *aureus* Newman and rendered the bacteria more susceptible to LL-37.

## Discussion

It has been established that an increased degree of resistance to human cathelicidin LL-37 is found in MRSA [16] and that the degree of LL-37 resistance among strains of pathogenic bacteria, such as group A streptococci, can correlate to the potential for invasive infection [17]. Thus, the identification and characterization of AMP resistance mechanisms in bacteria with pathogenic potential is of interest because the responsible genes and gene products could be promising targets for novel antibacterial agents. Our work described here demonstrates that BlaI renders *S*. *aureus* more resistant to the cathelicidin AMPs CRAMP and LL-37 as well as to whole blood killing. Additionally, our mouse challenge studies provide evidence that BlaI contributes to the virulence potential of beta-lactam resistant *S*. *aureus* strains *in vivo*.

It has been shown that MecI and BlaI bind to the same DNA binding motif and have co-regulatory effects on the expression of beta-lactamase and PBP2a: MecI can repress the BlaI target gene *blaZ* and BlaI can repress the MecI target gene *mecA* [18]. With respect to the similarities between MecI and BlaI, we speculate, that MecI could also contribute to the cathelicidin resistance and virulence potential of MRSA strains. The co-regulatory effects of MecI may also explain the diminished phenotypes seen in the MRSA252 background, which contains the *mec* operon, compared to the Newman background, which does not contain the *mec* operon.

Today, more than 95% of methicillin-susceptible *S*. *aureus* (MSSA) and MRSA isolates from human infections are resistant to penicillin [2]. It is commonly accepted that the bacteria acquired the BlaZ-BlaR1-BlaI-encoding genes through mobile genetic elements as a result of survival pressure following the introduction of penicillin as an anti-staphylococcal medication in the early 1940’s. Despite the fact that MRSA is resistant to beta-lactams—including beta-lactams susceptible to BlaZ hydrolysis—via expression of PBP2a (encoded by *mecA*), the majority of current, epidemic MRSA clones still express the BlaZ-BlaR1-BlaI-encoding locus [19]. The experimental observation that *blaI* contributes to staphylococcal resistance to host innate immune molecules and virulence extends the number of possible explanations for why so many MSSA and MRSA isolates harbor and keep the *blaZ*-*blaR1-blaI* genes: In addition to the continuing selection by beta-lactam antibiotics, the beta-lactamase regulatory system also might contribute to the fitness of *S*. *aureus* within the host by modulating the expression of virulence genes by BlaI. Heterologous expression of the BlaZ-BlaR1-BlaI system in beta-lactamase-negative *S*. *aureus* strains would allow to follow-up on this hypothesis.

Our study did not identify the exact mechanism(s) of action of how BlaI contributes to cathelicidin resistance and virulence in *S*. *aureus* Newman and MRSA252. It could not be attributed to the increased BlaZ beta-lactamase activity in the Newman *blaI* mutant, since the complemented Newman *blaI* mutant strain showed WT levels of cathelicidin resistance despite significantly increased BlaZ activity as compared to the parental strain. Hydrophibicity and surface charge also did not appear to play a role for the cathelicidin susceptibility of the *blaI* mutants. Despite the fact that *S*. *aureus* Newman WT and complemented *blaI* mutant strains had a markedly higher protease activity than the Newman *blaI* mutant on skim milk agar plates, no difference between the strains was found in terms of their capability to digest cathelicidin. From the results, it can be concluded that either the protease concentration in the *S*. *aureus* Newman supernatant was not high enough to observe cathelicidin degradation in the cell-free assays or a protease-independent mechanism might be responsible for the lower susceptibility of the WT strain to cathelicidin. In contrast, cathelicidin cleavage might contribute to the higher resistance of the MRSA252 WT strain to CRAMP. Follow-up studies comparing the resistance profile of the WT and *blaI* mutant strains in the presence of specific protease inhibitors would help further evaluate the role of proteases for the observed cathelicidin phenotype in *S*. *aureus blaI* mutants.

We speculate that the transcription factor BlaI can regulate genes beyond *blaZ* and *mecA*, which in turn leads to diminished cathelicidin killing of the bacteria. The above mentioned striking differences in protease activity between *S*. *aureus* Newman WT and *blaI* mutant on skim milk agar plates would support the hypothesis of a BlaI regulon that involves genes outside of the *bla* and *mec* operons. Evidence that regulators controlling beta-lactamase production may govern the expression of additional genes was shown for *Mycobacterium tuberculosis* [20]. Future studies may wish to conduct gene chip microarrays to identify differences in the gene expression patterns of *S*. *aureus* WT and *blaI* mutants. Moreover, putative BlaI binding sites in published *S*. *aureus* genomes may be found by searching for the conserved DNA motif TACA/TGTA in the promoter regions of open reading frames.

Finally, we showed that subinhibitory concentrations of 6-APA acid render beta-lactam resistant *S*. *aureus* more susceptible to innate host defense molecules, which could open an avenue of treatment regimes for difficult to treat *S*. *aureus* infections. The addition of beta-lactams to the treatment regimen in *S*. *aureus* infections to inactivate BlaI may render the bacteria more susceptible to cathelicidin-dependent host defense. Of note, beta-lactam treatment of MRSA can increase binding and activity of the cationic antibiotic daptomycin [21,22] suggesting exploration of such sensitizing mechanisms could prove a fruitful area for further investigation.

## Materials and Methods

### Ethics Statement

The animal experiments were carried out in strict accordance with the recommendations in the Guide for the Care and Use of Laboratory Animals of the National Institutes of Health. The Institutional Animal Care and Use Committee of the University of California, San Diego (Animal Welfare Assurance Number: A3033-01) approved all animal procedures prior to the experimentation. All efforts were made to minimize suffering of animals employed in this study.

Small volumes of blood for *in vitro* bactericidal assays were collected from healthy human volunteers under informed written consent through a protocol approved by the University of California, San Diego (UCSD) Human Research Protection Program IRB (#131002; protocol name: "Studies of Bacterial Resistance to Whole Blood or Neutrophil Killing”, P.I. V. Nizet)”.

### Bacterial Strains and Culture Conditions

The staphylococcal strains used for this study were *S*. *aureus* Newman and MRSA252 WT [5,12]. As a control for the screening of cathelicidin susceptible *S*. *aureus* Newman transposon mutants, an AMP susceptible *S*. *aureus* Newman *dltA* mutant [23] was used. For surface charge studies, the *S*. *aureus* ATCC-35556 (Sa113) WT and an isogenic *mprF* mutant strain, known to have an increased negative surface charge compared to the WT strain, served as controls [14]. *Tn*917 mutants and *blaI* and *blaZ* mutants were generated as described below, and 5–10 μg/mL erythromycin (Em) for *S*. *aureus* Newman [24] or 10 μg/mL chloramphenicol (Cm) for MRSA252 were employed for antibiotic selection. For complementation experiments in *S*. *aureus* Newman, 10 μg/mL Cm was used for culture of the WT and *blaI* mutant strains carrying the empty complementation vector pD123 or pDC123 with a copy of *blaI* (pBlaI). For production of knockout and complementation vectors, *Escherichia coli* strains were used as host and grown in Luria-Bertani broth (LB); the antibiotic selection employed 100 μg/mL ampicillin, 500 μg/mL Em or 10 μg/mL Cm. The restriction deficient intermediate host strain *S*. *aureus* RN4220 [25] was used to replicate plasmids produced in *E*. *coli* before transfer into the staphylococcal target strains.

### Preparation of *S*. *aureus* Newman Transposon Mutant Library

Random transposon mutagenesis of *S*. *aureus* Newman was performed using *Tn*917 essentially as described for Group B *Streptococcus* [13]. Briefly, the temperature sensitive suicide plasmid pTV_1_OK, which has a kanamycin (Km) resistance marker in the vector backbone and harbors *Tn*917 carrying an Em resistance gene, was introduced via electroporation into *S*. *aureus* RN4220. Plasmid DNA was isolated and transferred into the target strain *S*. *aureus* Newman. Several transformants were grown overnight in THB with 1 mg/mL Km at 30°C, a temperature permissive for pTV_1_OK replication. Overnight cultures were diluted 1/1,000 into THB without antibiotics, the temperature shifted to a non-permissive temperature (37°C) and incubated overnight yielding several potential random *Tn*917 mutant libraries which were stored at -80°C in THB, 35% glycerol. The randomness of *Tn*917 transposition in selected libraries was subsequently determined. To that aim, serial dilutions of the libraries were plated on Todd Hewitt agar (THA) plates with 5 μg/ml Em and incubated overnight at 37°C. The chromosomal *Tn*917 transposition events were identified in single colonies exhibiting Em resistance. The DNA of 10 randomly picked colonies per library was digested with *Hin*dIII and integration of *Tn*917 was probed for by Southern blot analysis using a digoxigenin-labeled transposon specific probe. The analysis of transposon mutant library #2 demonstrated a random chromosomal distribution of *Tn*917 insertion and a single integrated copy of *Tn*917 in at least 90% of the mutant strains (data not shown). For subsequent phenotypic screens, 4,800 transposon mutants of library #2 were picked, grown up overnight in 100 μl THB with 5 μg/ml Em and stored in a total of fifty 96-well plates at -80°C in THB + 40% glycerol.

### Screen for Cathelicidin Susceptible Transposon Mutants

A 96-well microtiter plate assay was established to screen transposon library #2 for mutants susceptible to the murine cathelicidin CRAMP. Notably, *S*. *aureus* is known to be highly resistant to cathelicidin AMPs in commonly used complex growth media such as THB or tryptic soy broth (TSB), but more susceptible in carbonate-containing solutions such as RPMI-1640 [26]. First, we showed that *S*. *aureus* Newman WT and the cathelicidin susceptible *S*. *aureus* Newman *dltA* mutant strain grew well in RPMI-1640, 10% LB medium. The minimal inhibitory concentration [13] of commercially synthesized CRAMP (Genemed Synthesis) in this medium was determined to be 3 μM for the *dltA* mutant, a concentration at which the WT strain was not inhibited. Subsequently, stationary phase cultures of the 4,800 *S*. *aureus* Newman *Tn*917 mutants were tested for their growth in the presence or absence of 3 μM CRAMP in order to identify genes that contribute to the cathelicidin resistance of the WT strain. Putative CRAMP susceptible candidate mutants identified in primary screens were subjected to additional testing to verify cathelicidin susceptibility. Their growth was compared to that of the WT strain in THB, and exponential phase mutant bacteria with no growth defect were re-tested for their cathelicidin susceptibility in CRAMP killing assays as described below.

### Identification of *Tn*917 Insertion Sites

The site of *Tn*917 insertion in CRAMP susceptible mutants was determined by using the following protocol. In a first PCR, genomic DNA obtained from the mutant strains was subjected to PCR with a random primer (5'-GGCCACGCGTCGACTAGTACATTACTAGCTACGCC-3') and the *Tn*917 specific primer 1st_left_*Tn*917 (5'-CCATGTTAAACCCATAGATAA-3'). The resulting amplification products were purified using standard procedures and subjected to a second and third PCR using the random primer mentioned above and the *Tn*917 specific primer 2nd_left_*Tn*917 (5'-ACACCTGCAATAACCGTTACC-3'). The binding site of 2nd_left_*Tn*917 is located downstream of 1st_left_*Tn*917 of the *Tn*917 sequence and thus was expected to specifically react with primary PCR products containing parts of the *Tn*917 and the disrupted Newman gene sequence. The final PCR products were purified and sequenced at Eton Bioscience Inc. (San Diego, CA) using the sequencing primer 3rd_left_*Tn*917 (5'-AGAGAGATGTCACCGTCAAG-3'). The obtained sequences were compared with published gene sequences in the GenBank database using the BLAST algorithm. In the transposon mutant G2E3, the insertion of *Tn*917 in *blaI* was verified by two PCRs using the primer *blaI*-integ (5-AGATCTTGTGTTGGGTTATTGAACA-3') in combination with either 1st_left_*Tn*917 or 2nd_left_*Tn*917.

### Targeted Mutagenesis

Plasmid insertional mutagenesis was performed to inactivate *blaI* in *S*. *aureus* Newman and MRSA252 as described [24]. For generating the *blaI* mutants, genomic DNA preparations from Newman and MRSA252 were used as templates. The primers *blaI_*ko_f_*Xba*I (5’-GCTCTAGATGGCCAATAAGCAAGTTGAA-3’) and *blaI_*ko_r_*Bam*HI (5’-CGGGATCCACTAATATCATTTAAAATGTC-3’) with overhangs constructing *Xba*I and *Bam*HI restriction sites into the PCR product were employed to amplify the first 351 bps of *blaI* by PCR. Please note that the *blaRI* gene, upstream of *blaI*, would be left intact using the described knockout strategy. The PCR products were cloned into pCR2.1-TOPO (Life Technologies, Grand Island, NY) according to the manufacturer’s instructions and propagated in *E*. *coli*. The plasmids were isolated, and the knockout constructs were digested with *Bam*HI/*Xba*I. For knocking out *blaI* in *S*. *aureus* Newman, the constructs were then ligated with *Bam*HI/*Xba*I digested temperature sensitive suicide vector pHY304, which confers Em resistance. The knockout vectors where then propagated in *E*. *coli*, subsequently transferred into *S*. *aureus* RN4220 and finally into the target strain Newman by electroporation using standard methods. The target strains carrying the knockout plasmids were grown under Em selection at 30°C, a temperature permissive for pHY304 replication. The cultures were shifted to a non-permissive temperature to allow only for survival of mutants with integrated plasmids in the presence of Em. PCR analysis for single Em resistant colonies was used to confirm the targeted disruption of *blaI* in *S*. *aureus* Newman. The same cloning strategy was used for generating MRSA252 *blaI*- and beta-lactamase-deficient *blaZ* mutants. However, since MRSA252 is instrinsically Em resistant, the restriction enzyme digested PCR products were cloned into the suicide vector pVE6007 [27], which carries a Cm resistance gene, and subsequently utilized to knock out *blaI* and *blaZ* as described above for the pHY304 construct.

### Complementation Analysis

For complementation of *blaI in trans* in *S*. *aureus* Newman, *blaI* plus flanking DNA was PCR amplified from a Newman genomic DNA preparation using primers *blaI*_complementation_f_*Sph*I (5’-CGGCATGCGAAAAGTATGAACTGTATGG-3’) and *blaI*_complementation_r_*Bam*HI (5’-CGGGATCCGAGTCAAGCATAGTTTACA-3’); the PCR product was cloned directionally into the expression vector pDC123 [28] which confers Cm resistance, yielding plasmid pBlaI. pBlaI was propagated in *E*. *coli*, transferred into *S*. *aureus* RN4220 and finally the *S*. *aureus* Newman *blaI* mutant as described above. As control strains for comparative functional analyses, the empty expression vector pDC123 was also put into the *S*. *aureus* Newman WT and *blaI* mutant strains.

### Nitrocefin Test

Nitrocefin is a chromogenic cephalosporin used to determine beta-lactamase activity [29]. When cleaved, it changes from yellow to red in color. Bacteria were grown in complex media, then washed, photometrically adjusted to the desired CFU concentration in phosphate buffer and then incubated with 50 μg/ml nitrocefin (CalBiochem/EMD Millipore, Billerica, MA) and incubated at 37°C for 30 min in the dark. The absorbances of the samples at 490 nm (A_490_) were read to determine the level of nitrocefin cleavage. Increased absorbance values corresponded to increased beta-lactamase activity.

### Minimum Inhibitory Concentrations for Penicillin and Daptomycin

Penicillin (Sigma-Aldrich, St. Louis, MO) MIC testing was performed by microbroth dilution in accordance with CLSI guidelines [30]. MICs were performed in triplicate and repeated three times. Daptomycin MICs were determined by Etest (bioMérieux, Durham, NC) according to manufacturer’s instructions. An Etest strip for daptomycin was placed on the center of the plate, and plates were incubated at 37°C overnight.

### Cathelicidin Killing Assays

Mid-log or stationary phase bacteria grown in THB or TSB were washed and subsequently spectrophotometrically adjusted to 10^7^ CFU/ml in Dulbecco’s phosphate-buffered saline (DPBS) + 10% TSB + 50 mM NaHCO_3_. 225 μl were added to 25 μl of CRAMP or LL-37 (SynPep Corporation, Dublin, CA) and incubated at 37°C. At the indicated time points, 25 μl were removed, diluted in PBS and plated on THA for enumeration of CFU.

### Human Whole Blood Assay

Blood was drawn from healthy donors and 10^6^ CFU of mid-log phase bacteria in 10 μl of DPBS + 10% TSB were added to 990 μl heparinized whole blood in siliconized tubes. Tubes were placed on a rotisserie at 37°C for 1 h, at which point 25 μl were removed, diluted in ddH_2_O to lyse blood cells, and plated for enumeration of CFU.

### Murine Skin Abscess and Systemic Infection Models

Eight-week-old female CD-1 mice (Charles River Laboratories, Wilmington, MA) were shaved and treated with Nair for hair removal on the day prior to injection. Mice were injected subcutaneously with 5 x 10^7^ CFU of mid-log phase Newman or Newman *blaI* mutant bacteria suspended in 100 μl of DPBS + 0.5 μg/ml cytodex beads (Sigma-Aldrich). Lesion sizes were monitored for 7 days and their area in mm^2^ calculated. No unexpected deaths occurred during the skin infection experiments.

For systemic infection, eight-week-old female CD-1 mice per group were injected intraperitoneally with 1.3 x 10^7^ CFU (Newman) or 6 x 10^8^ CFU (MRSA252) of mid-log phase WT or *blaI* mutant bacteria suspended in 200 μl of DPBS + 5% gastric mucin (MP Biomedicals, Santa Ana, CA). Survival was monitored every 12 h for 3 d. As a humane end point, mice were immediately euthanized if they were found moribund during the monitoring period.

To minimize animal suffering and distress, all mice were monitored twice a day for lack of mobility, hunched posture or moribundity caused by the experimental infections. Such animals were immediately humanely euthanized as described below. Anesthetics were not used during induction of the experimental infections, since the duration of the subcutaneous and intraperitoneal injection procedures were very brief. Pain medications were not used for the experiments since it was not clear how these affect infection outcomes.

Mice in all animal experiments were humanely euthanized in a designated CO_2_ inhalation chamber; the gas flow was maintained for at least two minutes after apparent clinical death; death of these mice was verified by cervical dislocation.

### FITC-Labeled Poly-L-Lysine Binding

Poly-L-lysine (PLL) is a positively charged molecule used to measure surface charge. The assay was modified from a previously described method [31]. Briefly, overnight cultures were washed twice with HEPES (20 mM, pH 7.25) and suspended to an A_578_ of 0.3. The bacterial suspension was incubated with 1 μg/ml FITC-labeled PLL (Sigma-Aldrich) for 15 min at room temperature and subsequently washed. By flow cytometry, the degree of PLL binding, which inversely reflected the relative positive surface charge, was determined. A total of 10,000 events were recorded and analyzed using a BD FACSCalibur instrument and software (Becton Dickinson, Franklin Lakes, NJ).

### Hydrophobicity

The hydrophobicity of bacterial cells was tested using a modified version of the MATH (microbial adhesion to hydrocarbons) assay [32]. Stationary phase bacteria were resuspended to A_600_ = 1.0 in DPBS. 900 μL were added to 300 μl hexadecane in triplicate. Tubes were vortexed for 2 min and subsequently left to stand at room temperature for 35 min to allow separation of hydrophobic and aqueous layers. 25 μL were subsequently removed from the aqueous layer, diluted in PBS and plated to allow for enumeration of CFU.

### Proteolytic Activity and Cathelicidin Degradation

The *S*. *aureus* Newman WT harboring the complementation vector pDC123, the *blaI* mutant + pDC123 and the complemented *blaI* mutant strains were grown in THB, Cm 10 μg/mL, washed and adjusted to equal CFU concentrations in PBS. Subsequently, 10 μL of the bacterial suspensions were dropped onto THA skim milk plates containing 10 μg/mL Cm to compare the proteolytic activities. The agar plates were incubated for 24–72 h at 37°C. Areas of clearance around the colonies indicated secreted protease activity. Protease activity tests with the MRSA252 WT and *blaI* mutant strains were carried out using skim milk agar plates without Cm.

To assess cathelicidin degradation, supernatants from staphylococcal overnight cultures grown in TSB were filter sterilized through a 0.22 μm syringe-driven filter (EMD Millipore). 18 μl of filtered supernatants were incubated with 2 μl of CRAMP or LL-37 to give a final concentration of 16 μM CRAMP or 8 μM LL-37. Samples were incubated at 37°C for 24 h, mixed with 4x sample buffer and 10x reducing agent (Life Technologies), boiled for 10 min, loaded onto a 12% Bis-Tris gel (Life Technologies) and run at 120 V in MES running buffer. Gels were stained with SimplyBlue SafeStain (Life Technologies) and subsequently destained in H_2_O.

### Treatment with 6-Aminopenicillanic Acid (6-APA)

Bacteria were grown to mid-log phase in DPBS + 10% TSB and resuspended to 10^7^ CFU/ml in the same buffer. Bacteria were incubated alone or in the presence of a subinhibitory dose of 6-APA in siliconized tubes on a rotisserie for 1 h at 37°C. Subsequently, *S*. *aureus* Newman bacteria were incubated with 24 μM LL-37 or water in a 96-well round bottom plate shaking for 1 h at 37°C. Subsequently, the CFU concentrations were determined as above.

### Statistical Analyses

Nitrocefin tests were analyzed using one-way ANOVA with Bonferroni post-test. Cathelicidin kinetics were compared using repeated measures two-way ANOVA with Bonferroni post-test except for MRSA252 + CRAMP, which was analysed using Student’s unpaired *t*-test. The whole blood assay was analysed using one-way ANOVA with Bonferroni post-test. Lesion sizes for the murine abscess model were compared using the Student’s paired *t*-test. The murine intraperitoneal challenge survival curves were compared using the log-rank (Mantel Cox) test. Poly-L-lysine-FITC binding was compared using one-way ANOVA. Hydrophobicity was analyzed using one-way ANOVA (Newman) or Student’s unpaired *t-*test (MRSA252). All statistical tests were performed using GraphPad Prism version 5.0 (GraphPad Software Inc., San Diego, CA). *P* values <0.05 were considered statistically significant.
